# Developing a Simplified Consent Form for Biobanking

**DOI:** 10.1371/journal.pone.0013302

**Published:** 2010-10-08

**Authors:** Laura M. Beskow, Joëlle Y. Friedman, N. Chantelle Hardy, Li Lin, Kevin P. Weinfurt

**Affiliations:** 1 Center for Genome Ethics, Law and Policy, Duke Institute for Genome Sciences and Policy, Duke University, Durham, North Carolina, United States of America; 2 Duke Translational Medicine Institute, Duke University, Durham, North Carolina, United States of America; 3 Center for Clinical and Genetic Economics, Duke Clinical Research Institute, Duke University School of Medicine, Durham, North Carolina, United States of America; Dalhousie University, Canada

## Abstract

**Background:**

Consent forms have lengthened over time and become harder for participants to understand. We sought to demonstrate the feasibility of creating a simplified consent form for biobanking that comprises the minimum information necessary to meet ethical and regulatory requirements. We then gathered preliminary data concerning its content from hypothetical biobank participants.

**Methodology/Principal Findings:**

We followed basic principles of plain-language writing and incorporated into a 2-page form (not including the signature page) those elements of information required by federal regulations and recommended by best practice guidelines for biobanking. We then recruited diabetes patients from community-based practices and randomized half (n = 56) to read the 2-page form, first on paper and then a second time on a tablet computer. Participants were encouraged to use “More information” buttons on the electronic version whenever they had questions or desired further information. These buttons led to a series of “Frequently Asked Questions” (FAQs) that contained additional detailed information. Participants were asked to identify specific sentences in the FAQs they thought would be important if they were considering taking part in a biorepository. On average, participants identified 7 FAQ sentences as important (mean 6.6, SD 14.7, range: 0–71). No one sentence was highlighted by a majority of participants; further, 34 (60.7%) participants did not highlight any FAQ sentences.

**Conclusions:**

Our preliminary findings suggest that our 2-page form contains the information that most prospective participants identify as important. Combining simplified forms with supplemental material for those participants who desire more information could help minimize consent form length and complexity, allowing the most substantively material information to be better highlighted and enabling potential participants to read the form and ask questions more effectively.

## Introduction

Advances in genomic research are generating opportunities for progress toward the vision of “personalized medicine”—tailoring risk reduction, diagnosis, and treatment strategies to individual patients to improve health, prevent disease, and potentially reduce healthcare costs [1], [2]. A necessary component of such efforts is the development of key translational research tools [3], including well-characterized, disease-specific and population-based biospecimen banks [4].

Research involving biospecimens, however, raises important issues concerning informed consent. Although the collection, storage, and research use of biospecimens and data are typically thought to involve minimal risk [5], information must be conveyed during the consent process that can be complex or controversial. Examples include the use of biospecimens and data for future research that is unspecified at the time of consent; indefinite storage; ongoing medical record access; contact for future research; large-scale data sharing; development of commercial products; privacy and confidentiality protections; participants' access to research results; and the ability to discontinue participation.

At the same time, there have been continuing calls for consent documents to be simplified [6]–[8]. Consent forms in general have lengthened over time [9]–[13]. In addition, although nearly half the adult population of the U.S. cannot read at higher than an 8th grade level [14], few consent documents are written at less than a 10th grade level [9]–[12], [15]–[18]. Not surprisingly, studies have shown that many participants may not understand the information disclosed [9], [19]–[21].

Thus, with regard to biorepositories in particular, the National Cancer Institute's Office of Biorepositories and Biospecimen Research has recommended that “…a 1-page informed consent document outlining important issues and risks in straightforward language should be developed and implemented” [8]. In response to this challenge, we developed a simplified biobanking consent form comprising what we considered to be the minimum information necessary to meet ethical and regulatory requirements. We then gathered preliminary data from hypothetical biobank participants about whether the form included the information they would find most important to a decision about taking part in a biobank. Here we describe our form and the rationale for its content, as well as the results of preliminary testing with research participants. We conclude by outlining essential next steps in the development and implementation of a simplified biobanking consent form.

## Methods

Federal policy for the protection of human research subjects, known as the Common Rule [22], as well as the Health Insurance Portability and Accountability Act (or “Privacy Rule”) [23] mandate that consent forms be written in plain language that the subject can understand [24], [25]. To reduce the level of education required to understand our form and to improve its overall readability (Table 1), we followed basic principles of plain-language writing, such as choosing common, everyday words; writing in first person and in active voice; keeping sentences short, with one main idea per paragraph; using clear organization and format with descriptive headings; and ensuring adequate white space and margins [26].

**Table 1:** Consent form readability characteristics*.

Characteristic	Measure	Result
Readability	Flesch-Kincaid grade level	6.7
	Flesch reading ease	71.5
	Passive sentences	11%
Complexity	Sentences per paragraph	3.1
	Words per sentence	13.8
	Characters per word	4.3
Length	Characters	5322
	Words	1188
	Sentences	84
	Paragraphs	30
	Pages (excluding signature page)	2

*Not including title and signature page. Statistics calculated using tools available in Microsoft Word 2007 (Redmond, WA). Readability tests are based on the average number of syllables per word and words per sentence. The Flesch Reading Ease test rates text on a 100-point scale; the higher the score, the easier it is to understand. The Flesch-Kincaid Grade Level test rates text on a U.S. school grade level.

As described below, we incorporated into our form (Exhibit S1; see Appendix S1 for detailed annotations) the elements of information required by federal regulations, as well as those recommended by best practice guidelines for biobanking [5], [27]–[29].

### Purpose

Consent forms must contain a statement that the activity involves research and an explanation of the purpose of the research, including the reasons that health information is being collected. Meeting these requirements in the context of a biobank is challenging, because the specifics of the future research are not known at the time of biospecimen collection. Even so, best practice guidelines [27], [28] suggest that the type of research can be anticipated and described sufficiently—including explicit mention of the nature and purposes of genetic research—to satisfy federal regulations. Our consent form addresses these issues in the introductory text, as well as with a succinct statement of the motivation for a biobank under the heading “What Is the Purpose of This Research Project?”

Note that our consent form is based on a biobanking model in which (a) an Institutional Review Board (IRB)-approved protocol and informed consent are required for the collection and storage of specimens and data; and (b) researchers proposing to use the stored materials must submit separate information about their particular study to an IRB for a determination regarding the involvement of human subjects [30], [31], exemption [32], or a waiver of the requirement to obtain consent [33]. Because this model assumes that no studies take place under the biorepository protocol, our consent form deliberately uses the terms “research” and “storage project” rather than “study.”

### Procedures

Consent forms must include a description of the procedures to be followed and identify any procedures that are experimental. The collection and storage of biospecimens and data for future use does not involve experimental procedures, but does involve several steps. We sequenced this information in a generally chronological fashion to make it easy for subjects to follow [26].

#### Biospecimen collection

Our form describes collection of blood through a standard venipuncture procedure.

#### Information collection

The Privacy Rule and best practice guidelines [27] require that the explanation of procedures include a description of the health information to be collected. Our form delineates three sources of information that will be linked to the participant's biospecimen: a questionnaire about basic demographic information and family health history; ongoing access to the participants' medical records; and research data generated from analyses of the biospecimen.

#### Access by researchers

Our consent form is consistent with best practice recommendations [5], [27]–[29] that consent documents disclose that biospecimens and data will be shared with qualified researchers; describe the oversight mechanisms that will be used to ensure that the research is scientifically and ethically appropriate; and state that identifying information will be removed from samples and information provided to investigators.

#### Recontact for additional research

Our form follows best practice guidelines [27] by disclosing the possibility that participants may be contacted about taking part in additional research and providing the option for participants to choose whether they would be willing to be contacted. We included a limit on the frequency of such contact, which may be important in reducing participant burden [34].

#### Large-scale data sharing

Compared with the procedures described under “Access by Researchers,” this section of our consent form discloses sharing that may occur outside the direct control of the biobank, such as the submission of data from genome-wide association studies (GWAS) to federal repositories. Institutions that submit data to the GWAS repository must certify that the consent documents signed by participants are consistent with such submission and with subsequent sharing for research purposes [35].

### Duration

The Common Rule requires that subjects be informed of the expected duration of their participation. The Privacy Rule similarly requires a statement of how long health information will be kept, although an authorization expiration date of “none” or “end of the study” are permissible for research, including for the creation and maintenance of a research database or repository. The permanent storage we describe in our form is considered best practice for biorepositories [27], subject to sufficient resources and foreseeable research utility.

### Risks

Consent documents must provide a description of any reasonably foreseeable risks, including, when appropriate, a statement that there may be some risks that are currently unforeseeable. The risks associated with biobanking are not usually physical; rather, they concern the misuse of information, including clinical and other personal data associated with specimens, as well as results derived from research using the specimens. In accordance with best practice guidelines [5], [27], our form discloses possible consequences of such misuse, including discrimination affecting employment or insurance. Additionally, because large-scale data sharing occurs outside the control of the biobank, the risks associated with such sharing are described separately.

### Confidentiality Protections

The Common Rule mandates that consent forms describe the extent to which confidentiality of records identifying the subject will be maintained. The Privacy Rule further requires that prospective participants be told who will use protected health information. Because the potential harms associated with biobanking are primarily informational, our consent form reflects best practice [27], [28] by explaining that materials will be maintained in coded form, and by describing who will have access to identifiable information (a small number of biobank personnel) and who will not (e.g., researchers who use the stored materials for study). Our form also notes that there are laws against the misuse of genetic information [36] but, because DNA is itself a unique identifier, it also states that confidentiality cannot be guaranteed.

Although “confidentiality” is the technically correct term to describe the protection of information that has been entrusted to a biobank, we chose to use “privacy” in its colloquial sense for greater reading ease.

### Benefits and Costs

Consent documents must describe any benefits to the subject or to others that may reasonably be expected from the research. As noted in our form, direct personal benefit from biobank participation is unlikely; rather, the benefits accrue to society at large if research discoveries are translated into clinical and public health practice. As per disclosures expected under the Common Rule, our form states that taking part in the biobank entails no additional costs to participants or their insurance.

### Voluntariness and Alternatives

Federal regulations require a statement that research participation is voluntary and that persons can refuse without penalty or loss of benefits to which they are otherwise entitled. Prospective participants must also be informed about any appropriate alternatives, which, in the case of a biobank, is simply not to participate. Our form emphasizes these aspects in the introduction and in a section titled “What Are My Options?”

### Discontinuing Participation

According to the Common Rule, consent forms must include a statement that subjects may stop participating at any time [37]. Similarly, a statement concerning the individual's right to revoke the authorization in writing is required under the Privacy Rule [38].

Discontinuing participation in a biobank is complicated by the fact that samples and data may have already been transferred to investigators for specific studies. As per best practice guidelines [27]–[29], participants must be allowed to withdraw the *remainder* of their specimen (assuming it can be identified via a code), but samples and data that have been distributed do not necessarily have to be recalled. In addition to highlighting this limitation, our consent document alludes to options that could be offered via a form should a participant wish to withdraw, such as [39], [40]:

- No more contact (e.g., to update personal information or recruit for other research)
- No further medical record access
- Unlink (i.e., remove the link between the code number and identifying information)
- No further use (i.e., destroy any part of the specimen remaining)

### Questions

As required by the Common Rule, our form instructs participants who to contact if they have questions about the research or their rights as research subjects.

### Additional Elements

In addition to elements of information required by federal regulations, best practice guidelines recommend other topics that should be discussed with prospective biobank participants, including the possibility that commercial products could be developed and the availability of research results.

#### Commercialization

According to best practice guidelines [27], consent documents should employ clear and specific language to address the use of stored materials by private or for-profit entities and the possibility of research leading to the development of commercial products. Our form notes the possibility of commercial products in the opening paragraph, as well as in a statement about payments from any profits under “Are There Any Costs or Payments?” It also describes a range of researchers who might access the materials, including researchers from academic institutions, the government, and industry.

#### Research results

The Common Rule requires that consent forms should include, when appropriate, a statement that significant new findings developed during the course of the research which may relate to the subject's willingness to continue participation will be provided. Perhaps more directly relevant to biobanks, best practice guidelines [27] call for consent forms to state whether or not individual or aggregate results will be released to research participants. As recommended by other groups [5], [41], our form states that participants should not expect to receive individual results (except in very rare and narrowly defined circumstances) but that general news about studies being done through the biobank will be publicly available.

### Elements Not Included

We did not include the following information in our simplified form:

#### Research-related injury

Consent forms for research involving more than minimal risk must explain whether any compensation and/or medical treatment are available if injury occurs. However, collection and storage of biospecimens and data are not typically considered to entail risks greater than those ordinarily encountered in daily life or during the performance of routine physical exams [5], [42].

#### Number of subjects

According to the Common Rule, consent forms should note, when appropriate, the approximate number of subjects involved in the study. Although our form refers to “all the other people who take part,” the ongoing nature of a biobank makes stating a precise number difficult and perhaps less important to a decision about taking part.

#### Tiered consent

Best practice guidelines suggest that biorepositories might consider allowing participants to specify the types of research for which their specimens will be used via a tiered system of consent. This kind of choice is most easily implemented when biospecimens are originally collected for research on a specific condition. In such a case, a binary choice could be presented between consenting to future research that condition only, versus on other conditions as well. However, tiered consent may be inappropriate if the purpose of the biobank—as reflected in our form—is to provide biospecimens for a broad range of research, in which case providing participants with a list of potential types of research would be burdensome and uninformative [27]. Our form suggests other kinds of choices that participants could potentially be offered; if implemented, these would necessitate a robust system to ensure that participants' stated wishes are followed.

#### Certificates of confidentiality

Biorepositories may consider using Certificates of Confidentiality to help protect identifiable research information from forced disclosure [27]. When in effect, consent forms must contain appropriate language that describes the protections and limitations a Certificate provides [43], [44].

## Results

In addition to satisfying basic regulatory and best practice requirements using language that is easy to read, we believe the level of detail provided in a shorter, simpler consent form should be guided by what a “reasonable person” would want to know in order to make an informed decision. Thus, after devising our 2-page document (not including the signature page) as described above, we recruited a diverse group of diabetes patients from community-based physician practices in Durham and Kannapolis, NC. (IRBs for the Duke University Health System and the Carolinas Medical Center-Northeast Medical Center approved this research and all participants provided informed consent.) Half of those recruited (n = 56) were randomized to read the 2-page form, first on paper and then a second time on a tablet computer. (The other half was assigned to a group that read a longer form [45].) Participants were encouraged to use “More information” buttons, available in each section of the electronic version on the tablet computer, whenever they had a question or wanted to know more (Appendix S2). These buttons led to a series of “Frequently Asked Questions” (FAQs) that we developed based on the more detailed information available in a model biobanking consent form of traditional length (i.e., 6+ pages) [45]. Specifically, for each sentence in the longer form that was not already represented in the 2-page form, we devised a question that participants might ask to which that sentence was the answer. Through these FAQs, participants had access to every sentence they might plausibly see in a detailed form of traditional length.

Participants were asked to use the computer's electronic stylus to highlight specific sentences in the FAQs they thought would be important if they were considering taking part in a biorepository. Each participant received the following scripted instruction:

*If you look at the answers to any of the Frequently Asked Questions, we would like to know whether they contain any information that—in your opinion—would be very important to know about taking part in a biorepository. If so, please highlight the sentences that have information that would matter most to you, if you were thinking about taking part in a biorepository.*

In other words, we asked them, in essence, to identify information that we should consider adding back to our simplified form.

On average, these participants identified 7 sentences in the FAQs as important (mean number 6.6, SD 14.7, range: 0–71). No one sentence was highlighted by a majority of the participants; thus, no clear mandate emerged for any specific item of information to be reincorporated into the main body of the electronic consent form. Further, 34 (60.7%) did not highlight any sentences in the FAQs, which suggests that these participants felt the 2-page form already contained the most important information. Available evidence indicates that participants at least considered the other information; on average, they clicked on 5 of the 16 “More information” buttons (mean 4.7, SD 5.1, range: 0–16).

These preliminary results suggest that the simplified form we had developed contains the information that most prospective participants identify as important to a decision about taking part in a biorepository. Obviously, some information not contained in the 2-page form was selected as important by some participants. The 10 sentences most often selected concerned medical record access, ethics review of proposed studies, information about re-contacting participants about additional research, privacy risks, and participant access to individual research results (Appendix S3; many of these items have been incorporated into the current version of the form (Exhibit S1).

## Discussion

The President's Council of Advisors on Science and Technology [3] recommended the creation of an integrated national network of standardized biospecimen repositories as well as continued efforts aimed at developing a standard consent template for the collection and storage of specimens and data for future research use.

We produced our form in response to this and other calls for the simplification of consent documents. Although longer than the 1 page form recommended by NCI [8], we believe our form comprises the minimum information necessary to meet ethical and regulatory requirements. Further, it meets our goal of being easier to read (<7^th^ grade reading level) and the results of our preliminary studies suggest that it contains the information that most prospective participants identify as important.

Clearly, however, informational needs vary at an individual level; approximately one-third of our participants wanted more details and there was little agreement on which items of information should be added to our form. Although we incorporated those most commonly selected, adding back every item of information that any individual might find important would undermine the goal of developing a shorter, simpler document that focuses on the most substantively relevant information based on a reasonable person standard. One solution proposed by those advocating simplified consent is the use of supplemental materials [6]–[8]. This approach could help minimize the length and complexity of the consent form, allowing key elements to be better highlighted and enabling potential participants to read the form and ask questions more effectively, while still providing additional details for those who desire them.

As with any model consent materials, it is imperative that someone using a form like ours customize it to convey accurately the specifics of the research at hand. Regulations and best practices do not in every instance point to one policy option, and in these situations, the content of our form represents one possible approach supported in the literature. However, there may be other ethically acceptable approaches and the fact that we selected one as a “placeholder” in our efforts to develop a simplified form should not be construed as a policy recommendation. For example:

- NCI Best Practices [27] state the participants should be told “whether or not” they will have access to individual research results (Recommendation C.2.2.4). The language in our form sets a high threshold for such release, but some studies may set a different threshold—particular those that aim to study participants' reactions to receipt of results [46].
- Our form suggests that participants who wish to discontinue participation will be offered various levels of withdrawal, but some studies may choose not to offer any options other than destroying the remaining sample, or may not have maintained a link that would allow the sample to be identified.
- Our form depicts several optional choices for participants, but a particular study might be designed to offer only some or none of these choices.

These three examples represent a general principle that applies to every section: It is essential that each be carefully considered and modified as needed—ideally with an eye toward maintaining succinct and simple language—to ensure that it accurately represents that research for which the form will be used.

Three steps are vital for finalizing a simplified approach to informed consent for biobanking. First, IRBs and institutional legal counsel must agree as to the ethical and regulatory sufficiency of the simplified form, within the context of a complete informed consent process. Second, there is a critical need for research to evaluate the simplified form (as compared to a “traditional” length form)—again, within the context of a complete informed consent process—with respect to prospective participants' understanding of what they are being asked to consent to, their satisfaction with the amount of information received, and their preference for one kind of form or another. Third, further study is needed on the effective use of supplemental materials. As one example, the FAQs we developed were simply a means by which to present additional information to participants in our preliminary study for consideration—but it is possible similar FAQs might serve as a useful format for supplemental material. The effectiveness of this or other formats in promoting participant understanding and satisfaction, and the ways that supplemental materials should be incorporated into the informed consent process are important areas for future research.

## Supporting Information

Exhibit S1 Simplified biobanking consent form.
(0.05 MB DOC)

Appendix S1 Annotated simplified biobanking consent form.
(0.10 MB DOC)

Appendix S2 Frequently Asked Questions.
(0.08 MB DOC)

Appendix S3 Additional sentences participants most often selected as important in preliminary testing.
(0.07 MB DOC)
