# Supplementary material for: Developing a Simplified Consent Form for Biobanking
Source: PLoS One. 2010 Oct 8;5(10):e13302. doi: 10.1371/journal.pone.0013302 (PMC2951917; doi:10.1371/journal.pone.0013302)
Supplement: Appendix S1 — Annotated simplified biobanking consent form. (0.10 MB DOC) [file pone.0013302.s002.doc]

**Appendix S2. Annotated Simplified Biobanking Consent Form**

| Researchers are trying to learn more about cancer, diabetes, and other health problems. Much of this research is done using human tissue, such as blood. Through these studies, researchers hope to find new ways to detect, treat, and maybe prevent or cure health problems. Some of these studies may be about how genes affect health and disease, or how genes affect response to treatment. Some of them may lead to new products, such as drugs or tests for diseases.  We are asking you to let us store some of your blood and health information so they might be used in these kinds of studies. This is because you have been a patient of the Duke University Health System. | - Common Rule 45 CFR §46.116(a)(1) - Privacy Rule 45 CFR §164.508(c)(1)(iv) - NCI Best Practice C.2 - ISBER K2.200 - RAND 10.7 |
| --- | --- |
| You can take part in this storage project or not. This consent form gives information to help you decide. Please read it carefully and take all the time you need to make your choice. Be sure to ask us as many questions as you want. Everyone who takes part in research should know that:   - Taking part may involve some risks. - Taking part is voluntary. If you choose to take part, you can quit at any time. - No matter what you decide, now or in the future, it will not affect your medical care. | - Common Rule 45 CFR §46.116(a)(8) |
| **WHAT IS THE PURPOSE OF THIS RESEARCH PROJECT?** The purpose of the Biobank is to collect and store blood and health information so researchers can use them in future studies. | - Common Rule 45 CFR §46.116(a)(1) - Privacy Rule 45 CFR §164.508(c)(1)(iv) - ISBER K2.200 - RAND 10.7 |
| **WHAT IS INVOLVED?** If you agree to take part, we will ask you to sign this form. We will give you a signed copy to keep. Here is what will happen next: | - Common Rule 45 CFR §46.116(a)(1) - Privacy Rule 45 CFR §164.508(c)(4) |
| 1. We will get a blood sample from you. We will use a needle to draw about 3 tablespoons of blood from a vein in your arm. | - Common Rule 45 CFR §46.116(a)(1) |
| 2. We will get some information about you and your health.   - We will ask you for some basic information, such as your name, age, race, and family’s health history. We will contact you no more than once a year to update this information. - We will get some health information from your medical record. Examples include test results, medical procedures, images (such as X-rays), and medicines you take. We will use your medical record from time to time to update this information. - We will get research data from any studies done using your sample and information. | - Privacy Rule 45 CFR §164.508(c)(1)(i) - NCI Best Practice C.2.2.5 - RAND 10.3, 10.3, 10.5 |
| 3. We will store your sample and information in the Biobank, along with those from all the other people who take part. There is no limit on the length of time we will keep it. | - Common Rule 45 CFR §46.116(a)(1) - Privacy Rule 45 CFR §164.508(c)(1)(v) - NCI Best Practice C.1.3 |
| 4. We will let researchers use the materials stored in the Biobank for approved studies. Researchers from Duke, other universities, the government, and drug- or health-related companies can apply to use the materials. A science committee at the Biobank will review each request. There will also be an ethics review. We will not give researchers your name or any other information that could directly identify you. | - NCI Best Practice C.2.2.3, C.2.2.5, C.4.2, C.4.3 - ISBER K2.200, K2.300, ISBER K2.500 - NBAC Rec 4 - RAND 10.6, 10.8: 10.9 |
| 5. We may contact you in the future with offers to take part in other research. There will be a new consent process just for those studies. We will contact you about no more than two such studies per year. | - NCI Best Practice C.2.2.8 |
| 6. Some of your genetic and health information may be placed in scientific databases, along with that from many other people. Information that could directly identify you will never be included. Researchers who want to study the information must apply to the database. | - NIH GWAS Points to Consider (p.10) |
| **WHAT ARE THE POSSIBLE RISKS?** The most common risks related to drawing blood from your arm are brief pain and/or bruising.  There is a risk that someone could get access to the data we have stored about you. In some cases, it could be used to make it harder for you to get or keep a job or insurance. There are laws against the misuse of genetic information, but they may not give full protection. We believe the chance these things will happen is very small, but we cannot make guarantees.  There is a risk that someone could trace the information in a scientific database back to you. Even without your name or other identifiers, your genetic information is unique to you. We believe the chance that someone will identify you is very small. But the risk may grow in the future if people come up with new ways of tracing information. | - Common Rule 45 CFR §46.116(a)(2), §46.116(b)(1) - NCI Best Practice C.2.2.6 - NBAC Rec 10 |
| **HOW WILL INFORMATION ABOUT ME BE KEPT PRIVATE?** Your privacy is very important to us and we will make every effort to protect it. Here are just a few of the steps we will take: | - Common Rule 45 CFR §46.116(a)(5) - NCI Best Practice C.3.2.1 - ISBER K2.300 |
| - We will remove your name and other identifiers from your sample and information, and replace them with a code number. We will keep the list that links the code number to your name separate from your sample and information. Only a few of the Biobank staff will have access to the list and they sign an agreement to keep your identity a secret. | - Privacy Rule 45 CFR §164.508(c)(1)(ii), §164.508(c)(1)(iii) - NCI Best Practice C.2.2.5, C.4.4 - RAND 10.8 |
| - Researchers who study your sample and information will not know who you are. They must also sign an agreement that they will not try to find out who you are. | - ISBER K2.300, K2.500 - RAND 10.9 |
| - We will not give information that identifies you to anyone, except if required by law. Information that is shared outside Duke may no longer be protected by the federal privacy law called ‘HIPAA’. But it will be protected as described in this form and may be covered by other privacy laws. | - Privacy Rule 45 CFR §164.508(c)(2)(iii) |
| **WHAT ARE THE POSSIBLE BENEFITS?** You will not get direct benefit from taking part. The main reason you may want to take part is to help researchers make discoveries that might help people in the future. | - Common Rule 45 CFR §46.116(a)(3) |
| **ARE THERE ANY COSTS OR PAYMENTS?** There are no costs to you or your insurance. You will not be paid for taking part. If any of the research leads to new tests, drugs, or other commercial products, you will not share in any profits. | - Common Rule 45 CFR §46.116(b)(3) - NCI Best Practice C.2.2.3 |
| **WILL I FIND OUT THE RESULTS OF THE RESEARCH?** You should not expect to get individual results from research done using your sample. We will offer to tell you something we discover only if it is about a disease that is likely to cause early death if not treated. You can get general news about the kinds of studies being done through the Biobank at [URL]. | - Common Rule 45 CFR §46.116(b)(5) - NCI Best Practice C.2.2.4 - NBAC Rec 14 - RAND 10.10 |
| **WHAT ARE MY OPTIONS?** Taking part in Biobank is your choice. You can choose to take part or not take part. If you choose to take part, you can change your mind at any time. | - Common Rule 45 CFR §46.116(a)(4), §46.116(a)(8) - Privacy Rule 45 CFR §164.508(c)(2)(ii) |
| **WHAT IF I CHANGE MY MIND?** Just call [number] and let us know. We will send you a form so you can tell us in writing what you would like us to do with any of your blood that we have not already given out for study. | - Common Rule 45 CFR §46.116(a)(8), §46.116(b)(4) - Privacy Rule 45 CFR §164.508(c)(2)(i) - NCI Best Practice C.2.2.9 - ISBER K2.200 - RAND 10.9 |
| **WHAT IF I HAVE MORE QUESTIONS?** For questions about this project, contact [name], the Biobank Director, at [number]. For questions about your rights as a research participant, contact the Duke University Health System Institutional Review Board at (919) 668-5111. | - Common Rule 45 CFR §46.116(a)(7) |
|  |  |
| **CONSENT STATEMENT** |  |
| The project staff has explained to me the purpose of the Duke Biobank, the procedures involved, and the risks and benefits. I have asked all the questions I have now, and I know who to contact if I have more questions.  I voluntarily agree that my blood and information can be stored at the Duke Biobank. I understand it may be used in future research to learn about, prevent, or treat health problems. |  |
| In addition, I have made the optional choices marked below. I know that I can take still take part in the Duke Biobank, even if I answer ‘no’ to any of these options. | - NCI Best Practice C.2.2.7 - NBAC Rec 9 - RAND 10.8 |
| 1. Someone from the Biobank can contact me once a year to update my personal information.  YES ________ NO ________  (initials) (initials) |  |
| 2. Someone from the Biobank can use my medical record from time to time to get updated information about my health.  YES ________ NO ________  (initials) (initials) |  |
| 3. Someone from the Biobank can contact me with offers to take part in up to two other studies per year.  YES ________ NO ________  (initials) (initials) |  |
| 4. My genetic and health information can be released, with no direct identifiers, into scientific databases.  YES ________ NO ________  (initials) (initials) |  |
| ______________________________________ ___________  Signature of Subject Date | - Privacy Rule 45 CFR §164.508(c)(1)(vi) |
| ______________________________________ ___________  Signature of Person Obtaining Consent Date |  |

**Key:**

**Common Rule:** Code of Federal Regulations. Title 45, Part 46, Protection of Human Subjects, 2005. (<http://www.hhs.gov/ohrp/humansubjects/guidance/45cfr46.htm)>.

**Privacy Rule:** Code of Federal Regulations. Title 45, Parts 160 and 164, Standards for Privacy of Individually Identifiable Health Information; Final Rule, 2006. (<http://www.hhs.gov/ocr/privacy/hipaa/administrative/privacyrule/privruletxt.txt)>.

**NCI Best Practice:** National Cancer Institute. Best Practices for Biospecimen Resources, 2007. (<http://biospecimens.cancer.gov/global/pdfs/NCI_Best_Practices_060507.pdf)>.

**ISBER:** International Society for Biological and Environmental Repositories. 2008 best practices for repositories: collection, storage, retrieval, and distribution of biological materials for research. Cell Preserv Technol 2008;6:5-58.

**RAND:** Eiseman E, Bloom G, Brower J, et al. Case Studies of Existing Human Tissue Repositories. "Best Practices" for a Biospecimen Resource for the Genomic and Proteomic Era. Santa Monica, CA: RAND Corporation, 2003.

**NBAC:** National Bioethics Advisory Commission. Research Involving Human Biological Materials: Ethical Issues and Policy Guidance, Volume 1. Rockville, MD: National Bioethics Advisory Commission, 1999.

**NIH GWAS:** National Institutes of Health. Genome-Wide Association Studies (GWAS). NIH Points to Consider for IRBs and Institutions, 2007. (<http://grants.nih.gov/grants/gwas/gwas_ptc.pdf)>
