# Supplementary material for: Developing a Simplified Consent Form for Biobanking
Source: PLoS One. 2010 Oct 8;5(10):e13302. doi: 10.1371/journal.pone.0013302 (PMC2951917; doi:10.1371/journal.pone.0013302)
Supplement: Appendix S2 — Frequently Asked Questions. (0.08 MB DOC) [file pone.0013302.s003.doc]

**Appendix S3: Frequently Asked Questions**

Researchers are trying to learn more about cancer, heart disease, diabetes, and other health problems. Much of this research is done using human tissue, such as blood. Through these studies, researchers hope to find new ways to detect, treat, and maybe even prevent or cure health problems. Some of these studies may be about how genes affect health and disease.

We are asking you to let us store some of your blood so it might be used in these kinds of studies. This is because you have been a patient of the Duke University Health System.

It is your choice to take part in this storage project or not. This consent form gives information to help you decide. Please read it carefully and take all the time you need to make your choice. Be sure to ask us as many questions as you want.

Everyone who takes part in research should know that:

- Taking part may involve some risks.
- Taking part is voluntary. If you choose to take part, you can quit at any time.
- No matter what you decide, now or in the future, it will not affect your medical care.

**More Information #1**

**What is “tissue”?**

‘Tissue’ means materials from the body, such as blood. Other examples include urine, skin cells, hair, nail clippings, or a small piece of a tumor, organ, or bone.

**What are “genes”?**

Genes, which are made up of DNA, have all the information needed to build and operate a human body.

**Why would researchers want to study my blood?**

Researchers often study tissue from people who have health problems and from people who do not.

**What kinds of information will I find in this consent form?**

This form explains the purpose of the research, what it involves, the risks and benefits, other choices you have, and your rights as a participant.

**Can I talk to other people before I decide?**

We urge you to talk with your family, friends, and doctor before making your choice.

**If I don’t benefit from taking part, who will?**

Research is meant to gain knowledge that may help people in the future.

**WHAT IS THE PURPOSE OF THIS PROJECT?** The purpose of the Duke Biorepository is to collect and store tissue and health information so researchers can use them in future medical studies.

**More Information #2**

**What is a “biorepository”?**

A biorepository is a collection of samples and information. This collection is called the Duke Biorepository (or just “the Biorepository” in the rest of this form).

**Who runs the Duke Biorepository?**

The director of the Biorepository is [INSERT NAME].

**WHAT IS INVOLVED?** If you agree to take part, here is what will happen:

1. We will ask you to sign this consent form. We will give you a signed copy to keep.

2. We will get a blood sample from you. We will draw about 3 tablespoons of blood from a vein in your arm.

**More Information #3**

**What kinds of things will you get from my blood?**

From this sample, we may be able to extract things like plasma, serum, blood cells, DNA, and RNA.

**What is “DNA”?**

DNA is short for deoxyribonucleic acid. DNA stores information in the form of a code. Parts of DNA that have complete messages are known as ‘genes.’ Genes give the instructions for building all the proteins that make our bodies work. Genes carry information that is passed on to future generations.

**What is “RNA”?**

RNA is short for ribonucleic acid. RNA delivers DNA’s genetic message to the part of a cell that makes proteins. RNA gives some information about which genes are turned on or off at one point in time. RNA is not passed on from generation to generation.

3. We will get some information about you and your health.

- We will ask you for some basic information, such as your name, age, race, and family’s health history. We will contact you no more than once a year to update this information.
- We will get some health information from your medical record. We will use your medical record from time to time to update this information.
- We will also get research data from any studies at Duke University in which you take part.

**More Information #4**

**For how long will you contact me to update my basic information?**

This will happen for as long as your sample is stored in the Biorepository, unless you tell us not to.

**What if I don’t want to be contacted?**

You can tell us now or in the future not to contact you.

**What kinds of information will you get from my medical record?**

Examples include information about your health problems, lab results, medical procedures, and medicines.

**Why do you need to look at my medical record?**

This is because future researchers need to know if you have any health problems. They may also need to know about any treatments you have had and how well the treatments worked.

**For how long will you be using my medical record to update my health information?**

This will happen for as long as your sample is stored in the Biorepository, unless you tell us not to.

**What if I don’t want you to look at my medical record?**

You can tell us now or in the future not to look at your medical record.

4. We will store your sample and information in the Biorepository. There is no limit on the length of time we will keep it.

**More Information #5**

**Will my sample be the only one?**

We will keep your blood and information in the Biorepository along with those from all the other people who take part.

**How many people will take part in the Biorepository?**

We do not know exactly how many people will take part. We expect it will be many tens of thousands.

**How long will you keep my sample and information?**

We will keep them as long as they are useful, unless you decide to stop taking part or we close the Biorepository.

5. We will let researchers use the materials stored in the Biorepository for approved studies. We will not give researchers your name or any other information that could identify you.

**More Information #6**

**How will researchers get access to the samples and information?**

Researchers can apply to study the samples and information stored in the Biorepository. A research committee at the Biorepository will review each application.

**How do I know the studies will be ethical?**

An ethics review will also be done. This kind of review is to make sure that risks are minimized and that the rights and welfare of people who take part in research are protected.

**What happens if a study is approved?**

If a study is approved, a part of your blood and some information about your health might be given to the researchers, along with samples and information from many other people.

**Will you tell me every time my sample and information are used in a study?**

No, we will not notify you every time your sample and information are used in a study.

**Who will you let study the stored materials?**

Materials stored in the Biorepository will be used mainly by researchers at Duke University. Researchers from other universities, the government, and drug- or health-related companies can also apply to use them. We will only let skilled researchers study the samples and information.

6. We may contact you in the future with offers to take part in other research. We will make sure not to contact you more than twice a year.

**More Information #7**

**What would this other research involve?**

Some researchers might apply to do a study for which they would need to contact you. For example, they might want to ask you to fill out a survey or do a phone interview.

**Will researchers ever contact me directly?**

Someone from the Biorepository will contact you first. We will tell you about the study so you can decide if it is okay to give the researcher your name. If you agree, the researcher will then contact you to tell you more about the study.

**If I give the OK for a researcher to contact me, does that mean I have to take part?**

There will be a new consent process just for that study. You can decide then to take part or not take part.

**What if I don’t want to be contacted about other research?**

You can choose, now or later, that you do not want us to contact you about studies like these.

7. Some of your genetic and health information may be placed in scientific databases. Your name and other information that could identify you will never be included.

**More Information #8**

**What is the purpose of these scientific databases?**

In order to speed research, it is often helpful for researchers to share the genetic information they get from studying blood or tissue samples. Other researchers can then compare that information to the genetic information from people in other studies. By sharing information, researchers can learn even more about human health and disease.

**Who keeps these databases?**

There are many scientific databases where your information may go. Some are kept by Duke University, some are kept by the National Institutes of Health, and some are kept by private companies.

**Who can look at the information in the databases?**

Some of these databases can be used by the public. Others are restricted and can only be used by approved researchers.

**Will anyone who looks at the database know the information is mine?**

Nobody will know just from looking at a database that the information is yours.

**What if I don’t want my information to be in a database?**

Your genetic and health information would be released into scientific databases only if you agree.

**WHAT ARE THE POSSIBLE RISKS?** The most common risks related to drawing blood from your arm are brief pain and/or bruising.

There is a risk that someone could get access to the data we have stored about you. In some cases, it could be used to make it harder for you to get or keep a job or insurance. We believe the chance that this will happen is very small, but we cannot make guarantees.

There is a risk that someone could trace the information in a scientific database back to you. Even without your name or other identifiers, your genetic information is unique to you. We believe the chance that someone will identify you is very small. But the risk may grow in the future if people come up with new ways of tracing information.

**More Information #9**

**Are there any other risks to having my blood drawn?**

Infection, excess bleeding, clotting or fainting is also possible, but unlikely.

**Does North Carolina have laws against genetic discrimination?**

North Carolina law says employers cannot deny someone a job based on genetic information (§95-28.1A). It also says insurers cannot charge more or refuse to cover someone based on genetic information (§58-3-215).

**Do laws against genetic discrimination mean there is nothing to worry about?**

Although there are laws against the misuse of genetic information, they may not give full protection.

**How will you protect my privacy?**

There are more details about this in the section below called “How Will Information About Me Be Kept Private?”

**HOW WILL INFORMATION ABOUT ME BE KEPT PRIVATE?** Your privacy is very important to us and we will make every effort to protect it. Here are just a few of the steps we will take:

- We will remove your name and other identifiers from your sample and information, and replace them with a code number.
- We will keep the list that links the code number to your name separate from your sample and information.
- Only a few of the Biorepository staff will have access to the list and all Biorepository staff sign a pledge to keep your identity a secret.

**More Information #10**

**Are there laws that protect my privacy?**

Federal privacy rules give safeguards for privacy, security, and authorized access.

**Will you give anyone else information that identifies me?**

We will not give information that identifies you to anyone without your permission, except if required by law.

**How will you keep the samples secure?**

We will keep samples in locked freezers in locked buildings.

**How will you keep the information secure?**

We will keep health information and research data on secure computers. These computers have many levels of password protection.

**How will you keep the list that links the code number to my name secure?**

We will store this list on secure computers. These computers have many levels of password protection.

**Will researchers know who I am?**

Researchers who study your sample and information will not know who you are. We will give them only the code number and not any information that directly identifies you.

**How do you know researchers will keep the samples and information secure?**

Researchers must promise to keep the coded materials secure. They must also promise they will not try to find out who you are.

**Will you put research information in my medical record?**

Research records are separate from medical records. We will not place any information that we get or create as part of this project in your medical record.

**Who else might have access to my records?**

Your research record may be reviewed in order to meet federal or state rules. Reviewers could include, for example, people from a federal agency (such as the Food and Drug Administration) or the sponsor of a particular study. A reviewer who looks at your research record may also need to look at your entire medical record.

**What protections will I have if reviewers look at my records?**

If we give information to a reviewer, it is no longer protected by patient privacy rules (called ‘HIPAA’). But it will be protected by other federal privacy rules.

**WHAT ARE THE POSSIBLE BENEFITS?** You will not get direct benefit from taking part. The main reason you may want to take part is to help researchers make discoveries that might help people in the future.

**More Information #11**

**How will the Biorepository help people in the future?**

Some of the studies may lead to new products, such as drugs or tests for diseases.

**ARE THERE ANY COSTS OR PAYMENTS?** There are no costs to you or your insurance. You will not be paid for taking part. If any of the research leads to new tests, drugs, or other commercial products, you should not expect to share in any profits.

**More Information #12**

**Will I get paid if I am injured?**

If you are injured as a result of taking part in this project, you can get urgent medical care at [INSERT PLACE]. But there is no commitment by [LIST ENTITIES] to give monetary compensation or free medical care to you in the event of a project-related injury.

**Who should I call about a project-related injury?**

For questions about a project-related injury or to report a project related-injury, contact [INSERT NAME] at [INSERT NUMBER] during normal business hours. You can leave a message at this number after hours, on weekends, and on holidays.

**Will you sell my sample to anyone?**

Your sample will be used only for research and will not be sold.

**Could anyone make money from the samples and information?**

Research sometimes leads to discoveries that may one day have commercial value. For example, research could lead to new tests, drugs, or other medical products.

**What if I don’t want my sample and information used for commercial purposes?**

If you take part in this project, that means you agree to let the Biorepository make your sample and information available for these uses.

**Why don’t you plan to share any profits?**

Development of new products usually relies on the study of samples from hundreds or thousands of people, not on any one person.

**Who owns the samples and any discoveries?**

The Duke University Health System and/or the developers will assert all rights of ownership in the samples, as well as all rights arising from use of the samples.

**WILL I FIND OUT THE RESULTS OF THE RESEARCH?** You should not expect to get individual results from research done using your sample. You can get general news about the kinds of studies being done through the Biorepository at [INSERT URL].

**More Information #13**

**Why won’t I get individual research results?**

Researchers must study samples and information from many people over many years before they can know if the results have meaning.

**Will my doctor get the results?**

The results will not affect your care right now. We will not give the results to your doctor and we will not put them in your medical record.

**Could researchers discover something that my doctor doesn’t already know?**

There is a small chance that researchers could learn something about your health that you and your doctor did not know before. For example, they might find that you have a gene that is already known to increase risk for a disease.

**Will you tell me if you find out something serious about my health?**

We will offer to tell you a finding like this only if it is about a disease that is likely to cause early death if not treated.

**How will you let me know if you find out something serious about my health?**

We will send a letter by certified mail asking you to contact [INSERT NAME]. [INSERT NAME] will arrange a time for you to meet with him or another health care provider to go over the information. We will not give out serious findings over the phone or by mail.

**WHAT ARE MY OPTIONS?** The decision to take part in the Biorepository or not is up to you. If you choose to take part, you can change your mind at any time.

**More Information #14**

**What does it mean to “take part” in the Biorepository?**

Taking part in the Biorepository means letting us get and store a blood sample, along with some basic health information, for possible use in future research.

**Is annual contact required?**

- Letting us contact you no more than once a year to update your personal information is optional.

**Is medical record access required?**

- Letting us use your medical record from time to time to update your health information is optional.

**Is contact about more research required?**

- Letting us contact you no more than twice a year with offers to take part in more research is optional.

**Is putting information into scientific databases required?**

- Letting some of your genetic and health information be released, with no direct identifiers, into both publicly accessible and restricted scientific databases is optional

**If I want to take part, do I have to agree to everything?**

You can still take part in the Biorepository even if you do not agree to any of the optional parts.

**WHAT IF I CHANGE MY MIND?** Just call [INSERT NUMBER] and let us know. We will send you a form so you can tell us in writing what you want us to do.

**More Information #15**

**What if I don’t want you to contact me any more?**

You can tell us *“no more contact.”* This means we would no longer contact you to update your personal information or with offers to take part in more research. But we would still have your okay to keep and use the sample and information we already have. We would also still have your okay to use your medical record to get updated information about your health.

**What if I don’t want you to use my medical record any more?**

You can tell us *“no more access.”* This means we would no longer contact you or get updated information from your medical record. But we would still have your okay to keep and use the sample and information we already have.

**What if I no longer want the Biorepository to be able to identify me?**

You can tell us *“unlink.”* This means we would forever remove the link between the code number and your name. We would still have your okay to keep and use the sample and information we already have, but we would have no way to know that they are yours. We would also have no way to contact you or use your medical record.

**What if I want to quit altogether?**

You can tell us *“no further use.”* This means we would no longer give researchers your sample or information. We would destroy any part of your sample left in the Biorepository. We would keep the information we already have for audit purposes only. Please note that if we have already given out some of your sample and information for study, we cannot call them back. Also, we cannot destroy knowledge already gained from the study of samples and information. But we would not give researchers your materials for any more studies.

**Will there be any penalty if I change my mind?**

No matter what you decide, now or in the future, it will not affect your medical care. There will not be any penalty to you and you will not lose any benefits you would otherwise be able to get.

**WHAT IF I HAVE MORE QUESTIONS?** For questions about this project, contact [INSERT NAME], the Project Director, at [INSERT NUMBER]. For questions about your rights as a research participant, contact the Duke University Health System Institutional Review Board Office at (919) 668-5111.

**More Information #16**

**What if there is still something I don’t understand?**

You should feel free to ask any questions. Your questions should be answered clearly and to your satisfaction.

**Is there anyone else I can call with questions?**

You can also call [INSERT NAME], the Study Coordinator, at [INSERT NUMBER].

**What if it is after hours?**

You can leave a message at these numbers after hours, on weekends, and on holidays.
