# Supplementary material for: Developing a Simplified Consent Form for Biobanking
Source: PLoS One. 2010 Oct 8;5(10):e13302. doi: 10.1371/journal.pone.0013302 (PMC2951917; doi:10.1371/journal.pone.0013302)
Supplement: Appendix S3 — Additional sentences participants most often selected as important in preliminary testing. (0.07 MB DOC) [file pone.0013302.s004.doc]

**Appendix S4. Additional Sentences Participants Most Often Selected As Important in Preliminary Testing (N = 56)**

| **Section** | **Sentence Selected** | **n** | **(%)** |
| --- | --- | --- | --- |
| **We will get some health information from your medical record.** | |  |  |
| Examples include information about your health problems, lab results, medical procedures, and medicines.* | | 8 | (14.3) |
| You can tell us now or in the future not to look at your medical record. | | 6 | (10.7) |
| **We will let researchers use the materials stored in the Biorepository for approved studies.** | |  |  |
| An ethics review will also be done.* This kind of review is to make sure that risks are minimized and that the rights and welfare of people who take part in research are protected. | | 8 | (14.3) |
| **We may contact you in the future with offers to take part in other research.** | |  |  |
| There will be a new consent process just for that study.* | | 8 | (14.3) |
| **There is a risk that someone could get access to the data we have stored about you.** | |  |  |
| North Carolina law says employers cannot deny someone a job based on genetic information. | | 7 | (12.5) |
| It also says insurers cannot charge more or refuse to cover someone based on genetic information. | | 7 | (12.5) |
| Although there are laws against the misuse of genetic information, they may not give full protection.*^ | | 6 | (10.7) |
| **You should not expect to get individual results from research done using your sample.** | |  |  |
| We will offer to tell you a finding like this only if it is about a disease that is likely to cause early death if not treated.* | | 8 | (14.3) |
| We will send a letter by certified mail asking you to contact [name]. | | 6 | (10.7) |
| We will not give out the findings over the phone or by mail. | | 6 | (10.7) |

* Based on this input from participants, we added these sentences to our simplified form (Exhibit S1)

^ We chose this sentence to add to our simplified form because the first clause concisely captures the basic information conveyed in the other sentences that participants selected in this section about risk (the existence of laws against the misuse of genetic information),
